# Viaxl: A Solution of a Low-Cost Real-Time Visual Accelerometer Based on Laser Speckle Optical Flow Detection

**DOI:** 10.3390/s20247033

**Published:** 2020-12-08

**Authors:** Zhitian Li, Wuhao Yang, Xingyin Xiong, Zheng Wang, Xudong Zou

**Affiliations:** 1 The State Key Laboratory of Transducer Technology, Aerospace Information Research Institute, Chinese Academy of Science, Beijing 100081, China; ztli@mail.ie.ac.cn (Z.L.); whyang@mail.ie.ac.cn (W.Y.); xyxiong@mail.ie.ac.cn (X.X.); wangzheng02@aircas.ac.cn (Z.W.); 2 School of Electronic, Electrical and Communication Engineering, University of Chinese Academy of Sciences, Beijing 100049, China

**Keywords:** visual accelerometer, laser speckle, optical flow detection, non-contact measurement

## Abstract

Non-contact and non-destructive acceleration measurement is receiving considerable attention due to their low cost, flexibility, and simplicity of implementation, as well as their excellent performance in some emerging applications such as medical electronics applications, vibration monitoring, and some other special scenarios. In this paper, a visual accelerometer system based on laser speckle optical flow detection named Viaxl is proposed. Compared with the conventional non-contact acceleration measurement method based on a laser system, Viaxl has moderate and stable performance with the advantages of low cost and simplicity of implementation. Experiment results demonstrate that Viaxl, which consists of a commercial camera and a low-cost laser pointer, can achieve real-time, non-contact acceleration measurement, and confirm the basic system performance of Viaxl: a measurement nonlinearity better than 1.3%, up to 31 dB signal-to-noise ratio, and 1150 Hz theoretic bandwidth; this demonstrates the huge potential of Viaxl in a wide range of applications, and provides a new possible technical method for non-contact acceleration detection.

## 1. Introduction

Accelerometers are the most necessary and widely used sensors employed in inertial measurement; they have have witnessed remarkable progress in recent years, and are expected to be increasingly employed in diverse fields of industrial and consumer applications. Modern accelerometers are typically strap-down accelerometers, such as micro-electro-mechanical accelerometers (MEMS AXLs) that are based on the detection of capacitive, piezo-resistive, or natural frequency change of the proof mass attached to the moving object when it is accelerating. Although these accelerometers have seen successful adoption in various applications, the real-time acceleration measurement based on the strapped-down method limits the applications that require non-contact or nondestructive measurement in some special or extreme scenarios, because the classic accelerometer is difficult to apply in conditions of high temperatures or high pressure, or when the structure of the measured object is easily affected by the added mass, such as medical electronic applications such as laser heartbeat detectors [1], vibration monitoring of mechanics or infrastructure such as the tip-timing vibration measurement of aero engines, and so on [2,3,4]. This problem motivates the search for novel acceleration measurement methods, and particularly optical methods, such as full field laser interferometry based on an image acquisition system [5,6,7,8], and laser Doppler vibrometry based on a photodetector [9,10,11]. Remarkably, the non-contact accelerometer based on laser interferometry [5] can even realize real-time vibration detection with up to 30 kHz with 1 nm axial resolution, which proves that the comprehensive performance and technical maturity of these classic laser-based optical measurement methods have reached a dramatically high level. Unfortunately, these conventional non-contact measurement methods require complex optical components such as optical filters and fiber couplers, as well as a complex and precise optical path, which increases the implementation difficulty. Thus, developing a new method for non-contact and nondestructive acceleration measurement with low cost, flexibility, simplicity of implementation, and high performance can be attractive.

The laser speckle detection method is an upcoming non-contact tiny motion measurement technology [12], whose basic principle is to directly capture the high contrast granular speckle pattern formed by multiple reflections and the interference of coherent light that is projected on the surface of the measured object, based on the fact that the displacement of the speckle is highly related to the motion of the object [13]. Even if the object moves slightly, the captured speckle will show obvious displacement. Due to its advantages of a simple optical path, low power consumption and cost, and convenient debugging and operation, the laser speckle detection method has been applied in medical detection, sound detection, fine measurement and so on [12,14,15].

In this paper, Viaxl (visual based accelerometer), a real-time non-contact and nondestructive accelerometer system based on laser speckle optical flow detection, is proposed. In this paper we attempt to introduce a visual optical flow detection method into the application of non-contact acceleration detection for the first time. A set of Viaxl solutions including a fast capture and storage method, a real-time acceleration calculation algorithm based on laser speckle optical flow detection, and an optical flow data post-process is proposed and applied into the system. The complete set of the Viaxl hardware system is introduced in this paper, consisting of a common laser pen, a commercial camera with a zoom lens, and a personal computer. Experiment results demonstrate that Viaxl can achieve robust acceleration measurement with a nonlinearity of 1.3%, an up to 31 dB signal-to-noise ratio, and 1150 Hz theoretic bandwidth, thus achieving real-time, non-contact acceleration measurement using a commercial camera and a low-cost laser pointer. Viaxl has the following advantages, which indicates its huge potential in a wide range of applications:

(a) Non-contact measurement, no additional mass, no effect on the properties of the measured structure.
(b) Supports measurement of multiple points at the same time.
(c) Supports measurement of difficult positions and tiny structures.
(d) Not affected by the material properties and state of the tested structure.
(e) Supports remote measurement.
(f) It is low-cost and easy to apply.

This paper is organized as follows. The basic principles and system configuration of Viaxl are introduced in Section 2, Section 3 presents and discusses the experimental results, and the conclusion of this paper is presented in Section 4.

## 2. System Prototype

### 2.1. Acceleration Calculation Algorithm Based on Speckle Optical Flow Detection

**Figure 1:**
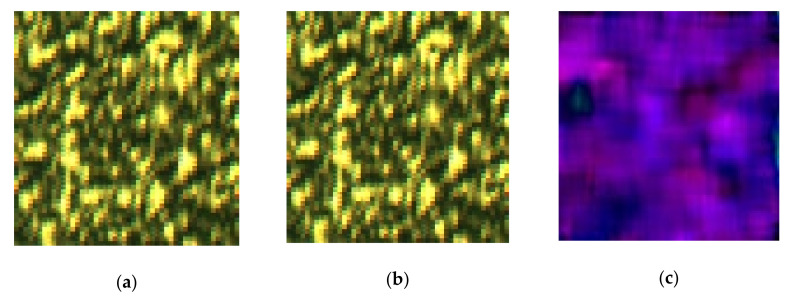
A group of adjacent speckle images (100 × 96 pixels) obtained by Viaxl and an optical flow diagram. (**a**) First frame; (**b**) second frame; (**c**) displacement between two adjacent frames (**a**,**b**), which was calculated by the acceleration calculation algorithm we propose.

In order to realize real-time and easy to use measurement, Viaxl does not use the traditional speckle capture and calculation method as in [14], where two speckle patterns are processed and superimposed to obtain the intensity-related fringe in a way similar to Young’s fringe, and then calculate the micro-displacement of the moving object. Viaxl uses a normal commercial camera to capture the laser speckle reflected by the moving object and calculate the ego-motion information between two adjacent frames of speckle through the optical flow algorithm, and then completes a real-time acceleration calculation. Due to the difficulty of acquiring the feature points in a speckle (Figure 1), Farneback optical flow—one of the most widely used dense optical flow algorithms—is used to estimate the displacement of the speckle [16]. The core idea of the Farneback algorithm is to estimate the polynomial approximation of speckle images using the neighborhood domain information of each pixel [17].

Acceleration calculation in Viaxl also begins by using the Farneback optical flow algorithm. Firstly, the captured speckle image is regarded as 2-dimensional gray level data $f\left(x,y\right)$ of pixel coordinate $X=\left(x,y\right)$. For each pixel of the speckle image, the local polynomial estimation is carried out according to the gray level of the pixel and the neighborhood information, and then we can construct the first polynomial:

(1) $$f_{1}\left(X\right)\sim X^{T}A_{1}X+b_{1}{}^{T}X+c_{1},$$

where $A=\left[\begin{array}{cc} r_{4} & r_{6}/2\\ r_{6}/2 & r_{5} \end{array}\right],b=\left[\begin{array}{c} r_{2}\\ r_{3} \end{array}\right],c=r_{1}\;$, $r_{1}\sim r_{6}$ are the coefficients of the polynomial $f_{1}\left(X\right)$ expansion. Assume that d is the displacement vector between adjacent speckle images, then we have Equation (2):

(2) $$f_{2}\left(X\right)=f_{1}\left(X-d\right)\;={\left(X-d\right)}^{T}A_{1}\left(X-d\right)+b_{1}{}^{T}\left(X-d\right)+c_{1}\;=X^{T}A_{1}X+{\left(b_{1}-2A_{1}d\right)}^{T}X+d^{T}A_{1}d-b_{1}{}^{T}d+c_{1}\;=X^{T}A_{2}X+b_{2}{}^{T}X+c_{2},$$

According to the assumption of invariable brightness, the corresponding grayscales of the polynomials $f_{1}\left(X\right)$ and $f_{2}\left(X\right)$ are equal, and thus the displacement vector of the image on sensor d can be obtained by Equation (3):

(3) $$d=-\frac{1}{2}A_{1}^{-1}\left(b_{2}-b_{1}\right),$$

Then we can calculate the real displacement of the object:

(4) $$D=dl\times \left(L-f\right)/f\;\propto d,$$

where l is the length of a single pixel of the image sensor, f is focus length, and L is the distance between object and image sensor. Figure 1 shows a group of adjacent speckle images obtained by Viaxl (100 × 96 pixels) and the corresponding optical flow diagram shown with the HSV (Hue, Saturation, Value) color gamut standard. In Figure 1, the tiny displacement between two adjacent frames can be seen intuitively.

Assume that the short-term displacement between adjacent speckle images is constantly accelerated; if we have obtained the timestamp difference of adjacent speckle images Δt, we can calculate the acceleration by numerical differentiation as in Equation (5):

(5) $$a=d^{2}D/d{\left(\Delta t\right)}^{2}\propto d,$$

It can be seen from Equation (5) that d is proportional to a, which proves that the value of d directly indicates the acceleration; thus we named the value of d the acceleration cardinal number (ACN). In real application scenarios, the coefficient between the acceleration and the ACN can be calculated by physical distance or obtained by pre-calibration.

To reduce the noise introduced in the calculation process, we use the optimized Farneback algorithm provided by OpenCV API (Application Programming Interface) to further improve the measurement accuracy.

### 2.2. Viaxl Prototype

**Figure 2:**
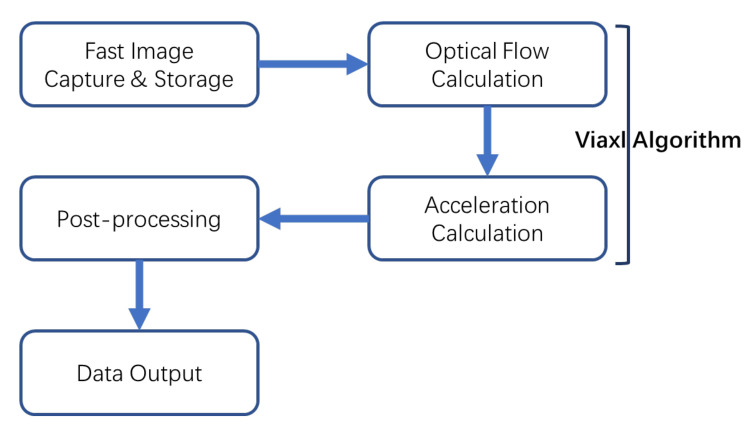
The workflow of Viaxl.

The Viaxl workflow contains four main parts: the camera of the Viaxl system captures the speckle images with a fast image capture and storage algorithm, and the speckle’s optical flow is calculated with the algorithm we proposed in Section 2.1. The results of the speckle’s optical flow are processed to prior-acceleration. Finally, the post-processing algorithm is introduced into the system and outputs the final acceleration data. Each part in Figure 2 will be presented in the following content.

#### 2.2.1. Fast Image Capture and Storage

**Figure 3:**
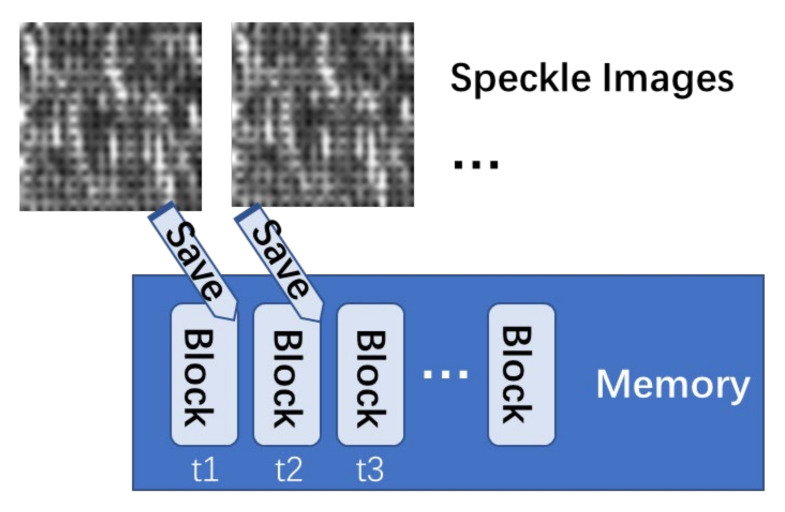
Diagram of fast image capture and storage process.

Considering the Viaxl real-time performance and measurement bandwidth requirements, an as high as possible image capture frame rate is essential. However, the original image capture software of normal commercial cameras cannot be used directly because of frame loss caused by insufficient memory, which will lead to a larger error in the acceleration calculation. Thus, a fast image capture and storage algorithm based on memory pre-partition is used, as shown in Figure 3: Firstly, according to the size of the speckle images to be captured, several blocks in the memory of the upper computer are designated for fast storage. After the image capture, the images are added to the designated memory block in turns, and the capture time is saved at the same time. The corresponding memory area will be released after the calculation for subsequent image capture and storage.

#### 2.2.2. Acceleration Calculation Based on the Viaxl Algorithm

The speckle images sequence captured by the camera is input into the Viaxl algorithm as we mentioned in Section 2.1, to calculate the prior acceleration of the object at the current time.

#### 2.2.3. Post-Processing

**Figure 4:**
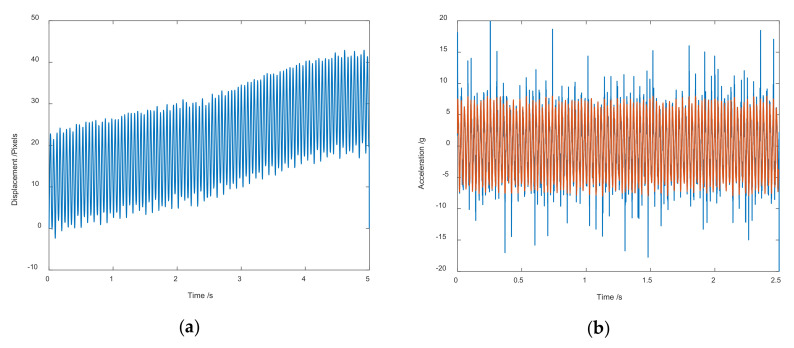
Results of post-processing: (**a**) Cumulative displacement of object motion calculated by Viaxl; (**b**) comparison of acceleration calculation with and without post-processing.

Because a CMOS image sensor is widely used in a common camera, random telegraph noise (RTS) is inevitable in CMOS. RTS will directly lead to random bright hot pixels in the image captured by the CMOS sensor, which has little effect in other ordinary image processing applications [18]. However, since the Viaxl algorithm calculates the acceleration based on the assumption of invariable brightness during the object’s motion, the size of the CMOS area and the aperture used in the Viaxl system is very small, and the image brightness is also low. Therefore, RTS will eventually cause cumulative errors and random errors of the motion displacement calculation by the optical flow algorithm. As shown in Figure 4, the cumulative error will be offset by the differential in the calculation of acceleration, but the random error is not eliminated. Considering that we do not particularly care about the high-frequency detail of the measurement, a moving window averaging algorithm is introduced in the post-processing part.

## 3. Experiment Results

### 3.1. Hardware System

**Figure 5:**
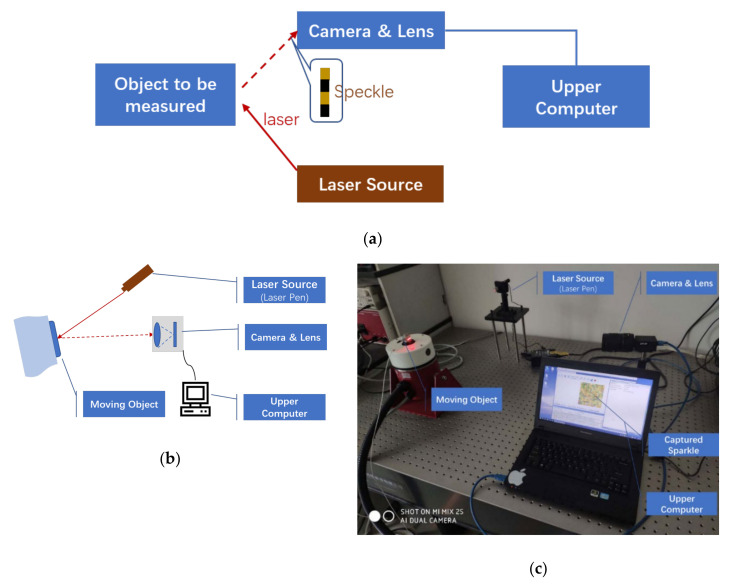
Hardware system and experiment scenarios of Viaxl: (**a**) Block diagram of Viaxl system; (**b**) system diagram of Viaxl hardware prototype; (**c**) experiment scenarios.

A basic Viaxl system with all hardware elements was designed to verify the performance of Viaxl, as shown in Figure 5. The basic hardware elements of a Viaxl contain a laser source, a camera, and an upper computer. Corresponding to the Viaxl system we designed, the specific devices used in each part are a FILR GS3-U3-23S6C-C camera with a 75 mm focal length lens, a Thorlabs CPS635F 635 nm laser diode module, and an MSI laptop with an Intel core i7 CPU. In order to ensure the instantaneity of calculation, the captured speckle image was set to 36 × 36 pixels, and the camera can achieve a frame rate of up to 2300 fps.

### 3.2. Experiment Results

To confirm the comprehensive performance of Viaxl directly, Viaxl was used to detect the acceleration of an adjustable frequency standard vibration platform (Data physics PA300E), and the results between the setup of the vibration platform and the output of Viaxl were compared.

#### 3.2.1. 10–100 Hz Acceleration Measurement Experiment

**Figure 6:**
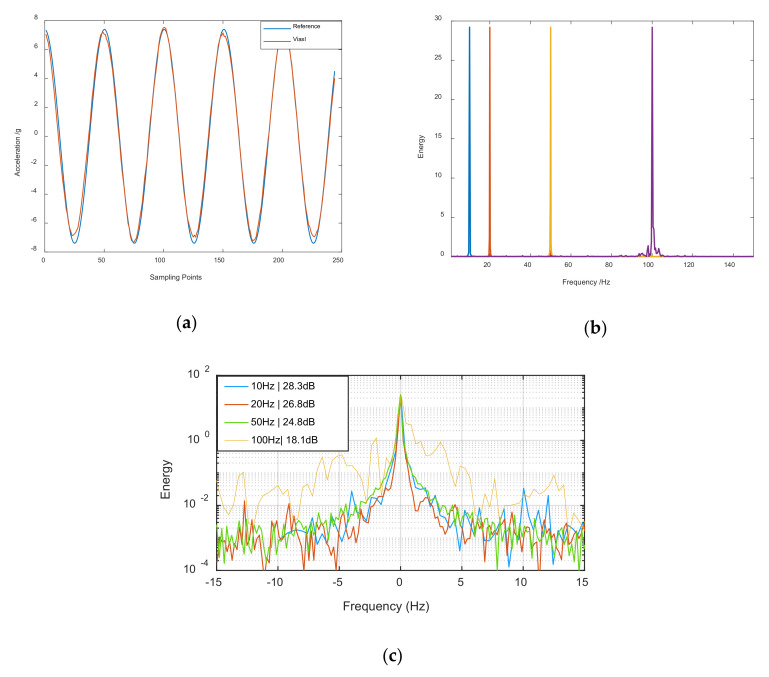
Accuracy of Viaxl’s results: (**a**) Comparison between the setting value of the platform and Viaxl’s result at 20 Hz; (**b**) spectra of 10–100 Hz Viaxl measurements; (**c**) comparison of power spectral density under different measurement frequencies (the abscissa is the difference between the actual vibration frequency and the measured vibration frequency).

Since the performance of a classic accelerometer is poor at low vibration frequencies, we paid more attention to performance under 100 Hz in the experiment. In the first experiment, the vibration frequency of the platform wax set to 10, 20, 50, and 100 Hz successively. The Viaxl real-time calculation results and the corresponding spectra (the spectrum is calculated by MATLAB since the core algorithm of Viaxl runs on a laptop) of each frequency are shown in Figure 6.

Figure 6a shows the comparison of acceleration between reference and Viaxl measurement (at 20 Hz); it can be seen that the Viaxl results were in good agreement with the reference. Figure 6b shows the spectrum of the Viaxl results under 10, 20, 50, and 100 Hz vibrations, and it can be seen that Viaxl showed good frequency accuracy and could complete the measurement task correctly under the different vibration frequencies. To observe the Viaxl measurements under different vibration frequencies more clearly, the spectra of Viaxl results under different vibration frequencies was further processed and is shown in Figure 6c, which demonstrates the noise performance of Viaxl when the Viaxl correctly calculated the frequency and acceleration of the real-time vibration of an object.

#### 3.2.2. Nonlinearity of Acceleration Measurement

**Figure 7:**
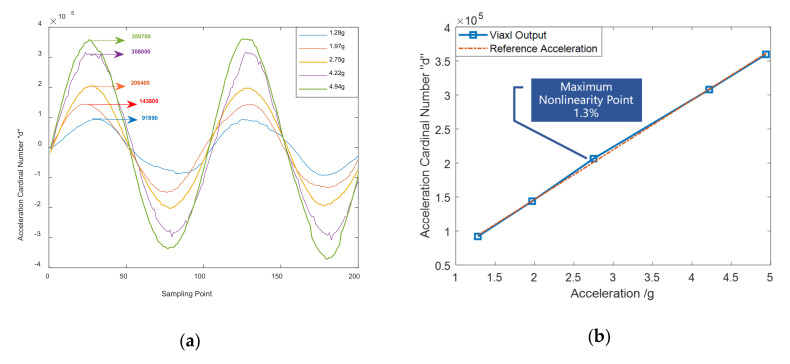
Nonlinearity verification of Viaxl at 20 Hz: (**a**) Time domain signal; (**b**) spectrum of 10–100 Hz Viaxl measurement.

In the interests of further verifying the acceleration measurement accuracy and stability of Viaxl, we conducted a system nonlinearity experiment according to the general performance verification method of classic accelerometers. In order to realize a convenient and reliable non-linearity experiment, the vibration frequency of the platform is left unchanged and the vibration amplitude is increased to change the real acceleration during the experiment. The maximum acceleration values of the vibration platform were set to 1.28, 1.97, 2.75, 4.22, and 4.94 g, and Viaxl with the same settings was used to measure the real-time acceleration of the platform. Comparing the ACN value obtained by Viaxl with the reference acceleration, the result shown in Figure 7 verified that the maximum nonlinearity of 1–5 g acceleration measurement obtained with Viaxl had a maximum nonlinearity point of 1.3% under the same setting, which proves the performance of Viaxl.

#### 3.2.3. Defocus Effect of Viaxl

The following experiment was used to explore the effect of defocus on Viaxl’s performance in the Viaxl system settings. In this experiment, the distance between the platform and the camera was fixed at 80 cm, and the focal length of the lens was calibrated as 0 mm when the camera is focused. On the basis of these settings, the focal length of the lens was increased to 15, 30, 45, and 60 mm, which caused an increasing defocus of the Viaxl system. The power spectral density under various focal length and corresponding signal-to-noise ratio (SNR) were then compared.

**Figure 8:**
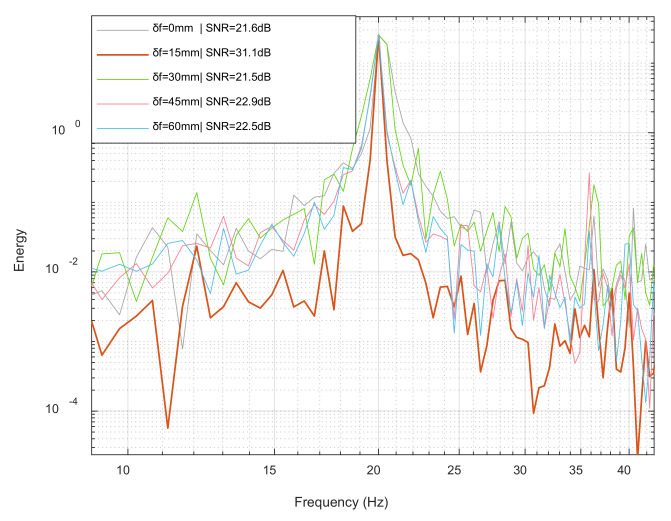
Power spectral density under 0–60 mm defocus lengths.

Figure 8 shows that with the increase of defocus, the noise performance of Viaxl first became better and then got worse. The potential reason is that proper defocusing will amplify the real-time vibration of the object to be measured, thus improving the SNR of the results. With the defocus increasing under the same conditions, the speckle is more sensitive to the motion of the measured object, and the displacement between the adjacent two speckle images will increase. However, the optical flow algorithm is not good at calculating too large of an image displacement, which results in a decrease of SNR.

#### 3.2.4. Minimum Detectable Amplitude

For sensors based on visual information, one of the biggest challenges is always the minimum detectable amplitude that can be supported. Viaxl can effectively enlarge the observed small vibration to the detection range of the optical flow detection algorithm. According to Equation (4), if the edge length of each pixel of the camera used in Viaxl is p, the minimum detectable vibration amplitude Dmin is according to Equation (6):

(6) $$Dmin=pl\times \left(L-f\right)/f$$

**Figure 9:**
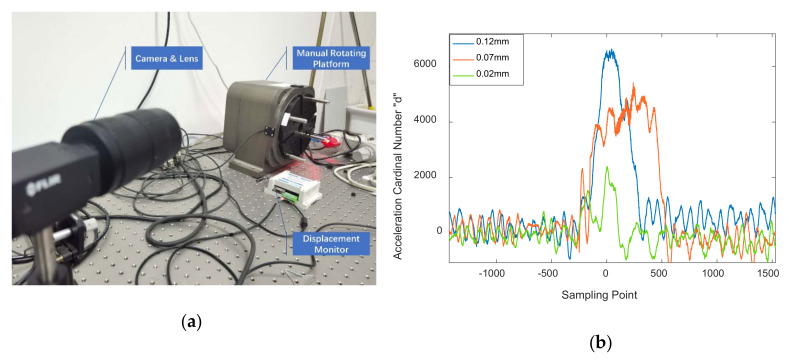
Minimum detectable vibration amplitude verification of Viaxl: (**a**) Single-axis high precision manual rotating platform; (**b**) experiment results.

To verify the minimum detectable vibration amplitude that Viaxl supports, as shown in Figure 9a, a single-axis high precision manual rotating platform with 0.3 μm movement resolution was used. The movement amplitude of the rotating platform was set to 0.12, 0.07, and 0.02 mm to verify that the minimum detectable vibration amplitude of Viaxl. Figure 9b shows the ACN values of Viaxl’s output at different vibration amplitudes. As shown in Figure 9b, the ACN values of Viaxl’s output had marked fluctuations when the vibration amplitude was greater than 0.07 mm, which is significant enough to be processed and calculated by the Viaxl algorithm. When the vibration amplitude was 0.02 mm, the ACN value was close to the background noise, which caused difficulty in calculating the real acceleration. The experiment result proves that the minimum effective supported detectable amplitude of Viaxl is at least 0.07 mm. This performance is enough to cover most non-contact acceleration measurement scenarios.

#### 3.2.5. Real-Time Performance and Maximum Bandwidth of Detectable Vibration Frequency

The maximum bandwidth of the detectable vibration frequency that Viaxl supports is related to the following two factors: the maximum frame rate of the camera used in the Viaxl system (which determines the theoretical maximum bandwidth) and the single elapsed time of the Viaxl algorithm in the processor used (which determines the real-time maximum bandwidth). In the Viaxl system proposed in this paper, the maximum frame rate of the camera used was 2300 fps (32 × 32 pixels), and the maximum bandwidth of Viaxl should be 1150 Hz according to Nyquist sampling theorem if the processor performance of the upper computer is powerful enough.

**Figure 10:**
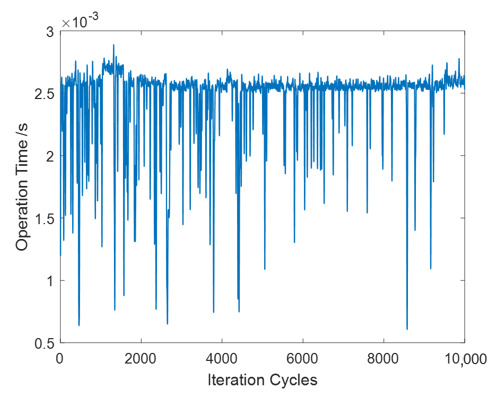
10,000 iterations of Viaxl elapsed time.

Compared with classic non-contact and non-destructive acceleration measurement methods, an advantage of Viaxl is better real-time performance at a lower cost, which means Viaxl is more suitable for fast acceleration and vibration measurement in consumer applications. As Figure 10 shows, 10,000 iterations of Viaxl elapsed time were recorded continuously to verify the real-time performance of Viaxl. It can be seen that the average Viaxl single elapsed time was about 2.5 ms, which means that the maximum real-time bandwidth of detectable vibration frequency limited by processor performance is 400 Hz.

## 4. Conclusions and Future Work

In this paper, a real-time non-contact and non-destructive acceleration measurement system named Viaxl based on laser speckle optical flow detection was proposed. Compared with other conventional methods, Viaxl calculates the acceleration by capturing the tiny displacement of the laser speckle that the measured object reflects so that it can obtain real-time acceleration information without attaching to the target object. Furthermore, the relationship between performance and the defocus degree of Viaxl was discussed. Experimental results show that the nonlinearity of real-time acceleration measurement was better than 1.3%, which proves the feasibility of the Viaxl system we presented for the real-time non-contact and non-destructive acceleration measurement with high accuracy and stability. More importantly, Viaxl, which is based on low-cost components, greatly reduces the implementation difficulty, which paves the way to the creative application of the non-contact and non-destructive accelerometer combining the simplicity of implementation and high performance.

As a first attempt of non-contact accelerometer implementation based on the laser speckle optical flow detection, the Viaxl presented in this paper can be improved in terms of bandwidth and accuracy through the hardware upgrades, such as using a camera with a high processing speed and frame rate [16]. On the other hand, improving the core algorithm of optical flow detection, such as in the waveform reconstruction of the time domain and estimation of moving direction, which have been preliminarily verified in [13,19], will greatly expand the application possibilities of the Viaxl system. More in-depth study for the system’s improvement is the subject of ongoing research on which we will report in a forthcoming publication.
